# Multimodal imaging of combined hamartoma of the retina and retinal pigment epithelium associated with an acquired vitelliform lesion

**DOI:** 10.1186/s40942-015-0023-6

**Published:** 2015-12-07

**Authors:** Bora Chae, Elona Dhrami-Gavazi, Kunal K. Dansingani, K. Bailey Freund, Winston Lee, Lawrence A. Yannuzzi

**Affiliations:** 1 Vitreous-Retina-Macula Consultants of New York, 460 Park Avenue, Fifth Floor, New York, 10022 NY USA; 2 The LuEsther T. Mertz Retinal Research Center, Manhattan Eye Ear and Throat Hospital, New York, NY USA; 3 Department of Ophthalmology, New York University School of Medicine, New York, NY USA; 4 Department of Ophthalmology, Edward S. Harkness Eye Institute, College of Physicians and Surgeons, Columbia University, New York, NY USA

**Keywords:** Optical coherence tomography angiogram, Combined hamartoma, Vitelliform lesion, Vitreo-macular traction

## Abstract

**Background:**

We present a case of a combined hamartoma of the retina and retinal pigment epithelium associated with a subfoveal acquired vitelliform lesion induced by vitreomacular traction. The purpose of this report is to present a unifying hypothesis of these concurrent findings, as aided by multimodal imaging.

**Case Presentation:**

A 25-year-old white man presented with a 6-month history of a visual disturbance in his left eye. At presentation, ophthalmic assessment showed a combined hamartoma adjacent to his optic nerve that had caused marked corrugation within the inner retinal surface. An acquired vitelliform lesion was present in the macula with an associated epiretinal membrane as demonstrated on spectral-domain optical coherence tomography. Optical coherence tomography angiography corroborated the clinical diagnosis of combined hamartoma.

**Conclusions:**

We are not aware of previous cases of a combined hamartoma associated with an acquired vitelliform lesion. As previously proposed in acquired vitelliform lesions related to epiretinal membrane and vitreoretinal traction, we believe that macular tractional forces might interfere with retinal pigment epithelium phagocytosis of shed outer segments, leading to the occurrence of this acquired vitelliform lesion.

## Background

Combined hamartoma of the retina and retinal pigment epithelium (henceforth referred to as combined hamartoma) was first described by Gass, in 1973, as an uncommon tumor in a series of seven patients [1]. He characterized these intraocular tumors in terms of thickened retinal tissue with a hyperpigmented tumor base and often presenting with contractions at the inner retinal surface [1]. He also concluded that they were congenital. Hisopathologically, combined hamartomas have been described as conglomerations of disorganized retinal, glial and vascular tissue intertwined with tubules of retinal pigment epithelium (RPE) with reduplication of the normally singular RPE layer [2, 3]. Several case reports have documented the association of these lesions with concurrent intraocular or systemic comorbidities such as neurofibromatosis types II and I [4–6], branchio-oculofacial syndrome [7], Gorlin-Goltz syndrome [8], Poland abnormality [9] and juvenile nasopharyngeal angiofibroma [10].

In the largest studied series of combined hamartomas (79 eyes from a single institution) Shields et al. found that common clinical characteristics included intralesional corkscrew vessels (65 %), retinal traction (81 %), and epiretinal membrane (61 %) [11]. According to Shields et al., the time-domain optical coherence tomography findings of combined hamartomas were most notable for dense epiretinal membranes leading to significant vitreoretinal traction with eventual retinal thickening and disorganization over the tumor itself [12]. Novel imaging techniques such as spectral-domain optical coherence tomography (SD-OCT) have allowed for better delineation of tumor tissue composition over the vitreoretinal interface. For instance, using enhanced depth imaging optic coherence tomography, Arepalli et al. described the presence of epiretinal membrane with traction in a sawtooth or folded pattern at the vitreoretinal interface [13]. While epiretinal membranes are known to occur commonly with combined hamartomas, we report a unique presentation of a combined hamartoma concurring with an acquired vitelliform lesion (AVL) in the macula, secondary to tractional forces. We further describe the intrinsic vascular pattern of a combined hamartoma using optical coherence tomography angiography (OCTA).

## Case presentation

**Fig. 1:**
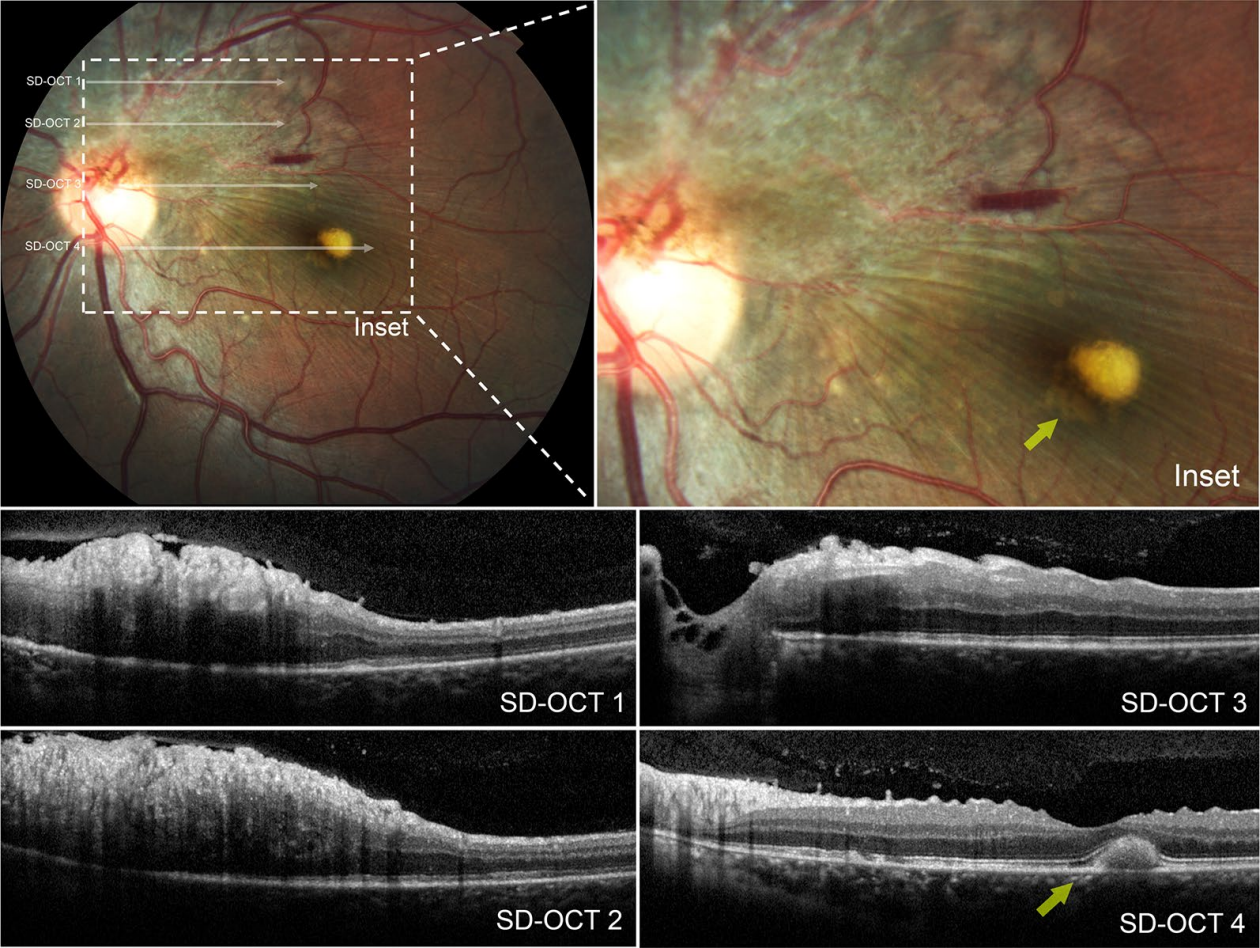
Color fundus photograph of the left eye in a 25-year-old man with a combined hamartoma, with magnified *insets* and accompanying horizontal cross sectional spectral domain optical coherence tomography (SD-OCT) images. A subfoveal vitelliform lesion is noted (*yellow arrow*)

A healthy 25-year-old white male presented complaining of a gradual and progressive visual disturbance in his left eye for 6 months. His Snellen best corrected visual acuity was 20/20 in each eye and the anterior segment examination was unremarkable bilaterally. Dilated fundoscopy of the right eye was normal; fundoscopy of the left eye showed a slightly elevated greyish lesion with scattered pigmentary changes superior to the optic nerve which extended in a fan-like distribution from the optic nerve (Fig. 1). There were tortuous fine retinal vessels overlying the lesion with radial retinal folds that appeared to be emanating from the infero-temporal aspect of the lesion. The folds extended into the macula and a small intraretinal hemorrhage was noted at the superior macula along with a vitelliform lesion near the fovea.

SD-OCT through the tumor showed thickening and disorganization of the inner and outer retina with marked saw-tooth like corrugations along the vitreoretinal interface (Fig. 1). The subfoveal vitelliform lesion appeared hyperreflective with preservation of the external limiting membrane and ellipsoid layer at the site of the lesion. SD-OCT also revealed the presence of an epiretinal membrane with contraction of the inner retinal layers of the macula.

**Fig. 2:**
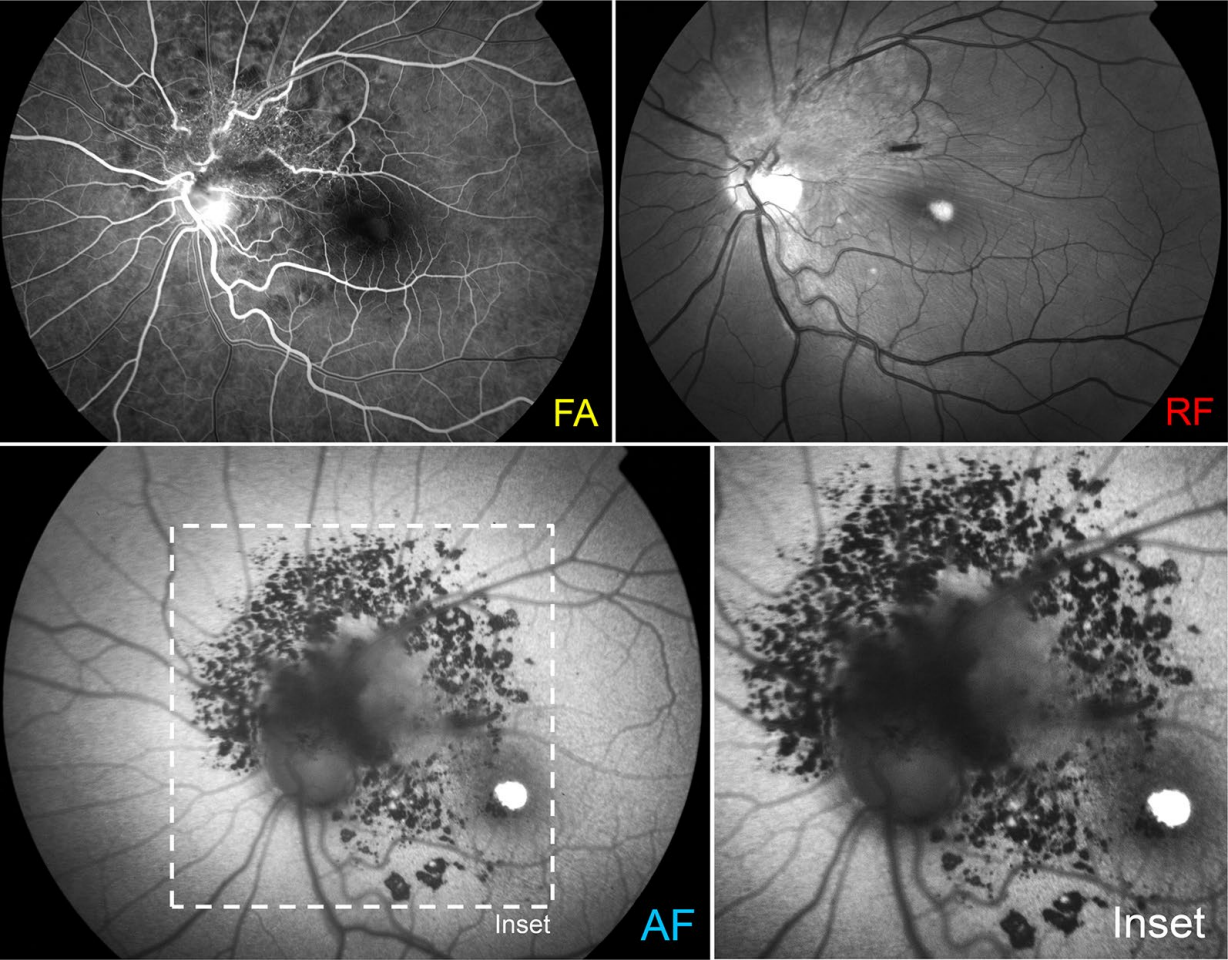
Multimodal imaging of the left eye of a 25-year-old man with a combined hamartoma and subfoveal vitelliform lesion. Fluorescein angiography shows distortion of the peripapillary vasculature while demonstrating the microcirculation within the hamartoma. Red-free imaging shows the extent of tangential traction with radial striae emanating from the tumor, which involves the fovea. Fundus autofluorescence shows speckled pattern of hypoautofluorescence due to the masking effect of the hamartoma. The subfoveal vitelliform lesion is intensely hyperautofluorescent

Fundus autofluorescence showed intense hyperautofluorescence from the vitelliform lesion as well as granular hypoautofluorescence inferotemporal to the lesion (Fig. 2). A linear masking of the background autofluorescence was visible in the area of the intraretinal hemorrhage. While retinal autofluorescence was obscured at the superior optic nerve head margin, a notable, large, speckled pattern of hypoautofluorescence was visible surrounding the optic disc and extending well beyond the clinically visible boundaries of the lesion along its entire perimeter. Early hyperfluorescence of the combined hamartoma was visible in fluorescein angiography along with an increased intrinsic vascularity within the lesion itself (Fig. 2).

**Fig. 3:**
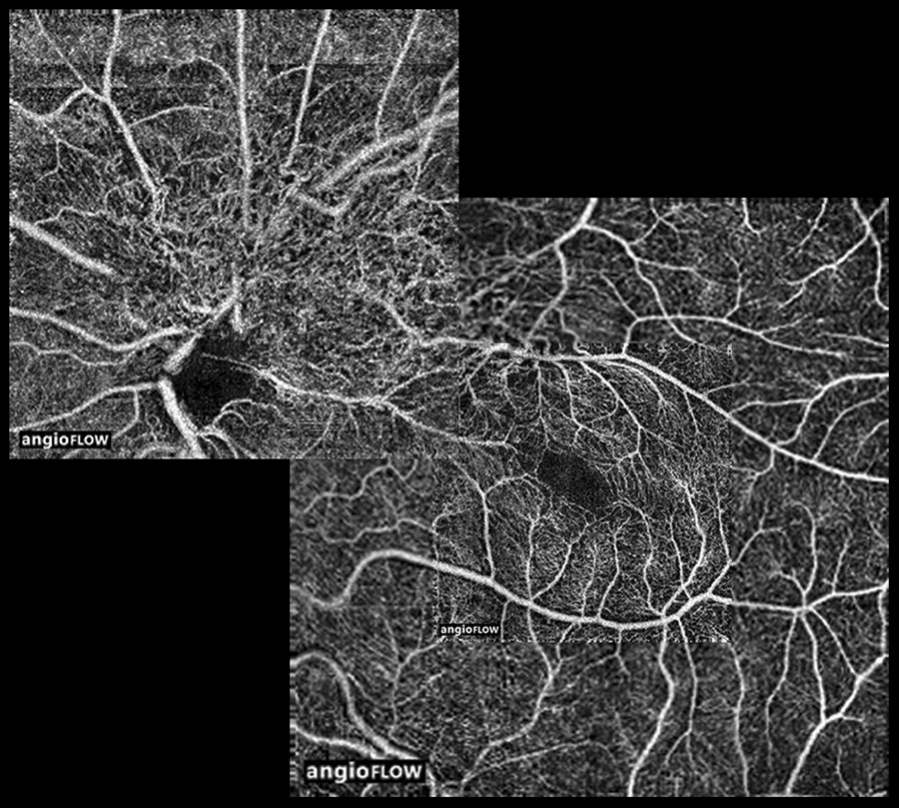
En-face optical coherence tomography angiography (OCTA) montage of the left eye of a 25 year old man with a combined hamartoma and subfoveal vitelliform lesion incorporating a 4.5 × 4.5 mm scan of the peripapillary area, a 6 × 6 mm scan of the macula, and a higher resolution 3 × 3 mm scan of the fovea. Segmentation of all scans includes the full thickness of the neurosensory retina. The flow signature corresponding with the small vessels of the hamartoma has a disorganized morphology. Tractional effects and distortion of the foveal avascular zone are also appreciated

OCTA revealed significant distortion of the retinal microcirculation intralesionally along the radial peripapillary capillary network superior and superotemporally to the optic nerve head with vessels appearing fine and tortuous in a corkscrew pattern (Fig. 3). Surrounding this network of corkscrew vessels were straight radial vessels normally found in a physiological peripapillary capillary network.

Similar to the description of combined hamartomas by Gass in 1973 [1], our patient presented with a unilateral grey lesion that was slightly elevated, involving the RPE, retina and the overlying vitreous which extended in a fan-like projection towards the periphery and blended imperceptibly with the surrounding tissue. After the initial identification of combined hamartomas by Gass, many other studies have documented common manifestations observed with these tumors, including a study by Shields et al. [4]. In a similar study of 60 patients, combined hamartomas are described as having vascular tortuosity (93 %), pigmentation (87 %), elevation (80 %), vitreoretinal interface disturbances (78 %), and lipid exudation (7 %) [14].

These observed clinical characteristics are confirmed and further elaborated with the aid of multimodal imaging, including novel techniques such as OCTA, which allowed for depiction of the tumor’s vasculature without the need for intravenous dye injection and without the limits in resolution arising from extravasation of fluorescein dye [15]. OCTA confirms and topographically maps out the increased and seemingly disorganized vasculature in the tumor as previously described [3, 14]. While the presence of thickened glial and retinal tissue makes it difficult to view the vascular architecture on fundoscopic examination, OCTA isolates and enables clear visualization of the vascular network.

Our patient manifested many of the characteristics mentioned above. However, he presented uniquely with a subfoveal acquired vitelliform lesion in the setting of traction occurring at the vitreoretinal interface. Acquired vitelliform lesions are known to occur in tractional maculopathies in which significant vitreomacular traction is present [16, 17]. For instance, in a study of 87 patients with unilateral epiretinal membrane, a subfoveal vitelliform lesion was found in 15 patients (17.2 %) compared to only 5 patients (5.7 %) in matched control fellow eyes (p = 0.03) [17]. According to Freund et al., normal apposition between the photoreceptor tips and the RPE is disrupted by tractional forces which can interfere with phagocytosis of shed outer segments in the RPE [16]. Subsequent accumulation of lipid-laden macrophages and RPE cells may account for the yellow vitelliform collection which appears hyperreflective on SD-OCT [16]. The common natural progression of AVLs is gradual absorption of the lesion with subsequent atrophy or, in rare cases, formation of a macular hole; both scenarios lead to poor visual outcomes [18–20].

A combined hamartoma can cause visual impairment in several ways, but vision loss most commonly occurs when retinal dragging causes foveal distortion [11, 14, 21]. When significant visual impairment is observed secondary to fovea-involving traction, treatment options include surgery to relieve the vitreomacular tractional component. Surgical management, however, remains controversial, as previous reports documenting visual improvement following vitrectomy and membrane peeling are anecdotal [22–25]. While combined hamartoma is a benign tumor, macular edema and choroidal neovascularization (CNV) can occur in association with these lesions, which can lead to a poor visual outcome [26]. Treatment of CNV in the past has included photodynamic therapy and laser photocoagulation but, more recently, intravitreal antiangiogenic injections have been utilized [27, 28].

## Conclusion

Diffuse retinal folds seen emanating from the tumor and extending over the macula indicate the presence of significant macular contraction where the subfoveal vitelliform lesion lies. SD-OCT served as a diagnostic adjunct to further localize the area of traction occurring along the vitreoretinal interface as evidenced by the presence inner retinal corrugations with an overlying epiretinal membrane. AVLs have been noted to occur in tractional maculopathies caused by epiretinal membranes, and we believe that the tractional forces described above resulted in the formation of an AVL in our patient. We additionally attribute the epiretinal gliosis and subsequent traction on the inner retinal vessels to have caused the linear retinal hemorrhage seen in the superior macula.

Both combined hamartomas and AVLs are capable of compromising vision if the integrity of the ellipsoid is compromised, either through direct traction or due to late outer retinal changes often seen in acquired vitelliform lesions. Despite the significant retinal striations and contraction seen in our patient, his foveal architecture remained generally undisturbed with relative sparing of the outer retinal landmarks accounting for his preserved visual acuity. Furthermore, as our patient did not exhibit any evidence of edema or CNV in association with the combined hamartoma, there was no rationale for the use of anti-inflammatory agents or antiangiogenic injections. Given these findings and the lack of consistent favorable outcomes after surgical intervention, close observation was recommended to the patient.

In summary, the advent of novel imaging modalities including SD-OCT has allowed for detailed characterization of combined hamartomas, particularly at the vitreoretinal interface. We are not aware of previously published OCTA imaging of combined hamartomas, which, in our patient, revealed features of the tumor’s microvascular morphology hitherto only seen in histopathological studies.

## Consent

Written informed consent was obtained from the patient for publication of this Case report and any accompanying images.

## Abbreviations

RPE: retinal pigment epithelium
AVL: acquired vitelliform lesion
SD-OCT: spectral-domain optical coherence tomography
OCTA: optical coherence tomography angiography
CNV: choroidal neovascularization
